# Functional conservation of EXA1 among diverse plant species for the infection by a family of plant viruses

**DOI:** 10.1038/s41598-019-42400-w

**Published:** 2019-04-11

**Authors:** Akira Yusa, Yutaro Neriya, Masayoshi Hashimoto, Tetsuya Yoshida, Yuji Fujimoto, Naoi Hosoe, Takuya Keima, Kai Tokumaru, Kensaku Maejima, Osamu Netsu, Yasuyuki Yamaji, Shigetou Namba

**Affiliations:** 1 Laboratory of Plant Pathology, Department of Agricultural and Environmental Biology, Graduate School of Agricultural and Life Sciences, The University of Tokyo, Yayoi 1-1-1, Bunkyo-ku, Tokyo, 113-8657 Japan; 2 Present Address: Laboratory of Plant Pathology, School of Agriculture, Utsunomiya University, Mine-machi 350, Utsunomiya, Tochigi, 321-8505 Japan

## Abstract

Since the propagation of plant viruses depends on various host susceptibility factors, deficiency in them can prevent viral infection in cultivated and model plants. Recently, we identified the susceptibility factor *Essential for poteXvirus Accumulation 1* (*EXA1*) in *Arabidopsis thaliana*, and revealed that EXA1-mediated resistance was effective against three potexviruses. Although *EXA1* homolog genes are found in tomato and rice, little is known about which viruses depend on EXA1 for their infection capability and whether the function of EXA1 homologs in viral infection is conserved across multiple plant species, including crops. To address these questions, we generated knockdown mutants using virus-induced gene silencing in two Solanaceae species, *Nicotiana benthamiana* and tomato. In *N*. *benthamiana*, silencing of an *EXA1* homolog significantly compromised the accumulation of potexviruses and a lolavirus, a close relative of potexviruses, whereas transient expression of EXA1 homologs from tomato and rice complemented viral infection. EXA1 dependency for potexviral infection was also conserved in tomato. These results indicate that EXA1 is necessary for effective accumulation of potexviruses and a lolavirus, and that the function of EXA1 in viral infection is conserved among diverse plant species.

## Introduction

Since plant virus genomes encode a limited number of genes, viruses rely on the recruitment of host plant factors for several steps of infection, such as the translation of viral proteins, replication of viral genomes, cell-to-cell movement through plasmodesmata, and long-distance movement via the vascular system^1,2^. Over the past several decades, a number of critical host factors have been identified and functionally characterized to reveal the molecular mechanisms of viral propagation^3–5^. Because these host factors play essential roles in viral infection, their loss-of-function mutations frequently confer virus resistance upon plants^6^. These functional features suggest that such host factors, termed as susceptibility factors after their functional features conditioning virus susceptibility, are potential targets for disruption or functional suppression for the artificial development of virus-resistant crops^7–11^. To this end, extensive functional analysis of susceptibility factors is essential to determine their roles in diverse virus–plant interactions.

Plant antiviral resistance attributed to loss-of-function mutations in essential susceptibility factors is referred to as “recessive resistance”^12^. Naturally occurring mutations in *eukaryotic translation initiation factor (eIF) 4E* and *4G* and their isoforms (eIFiso4Es) are widely used to induce recessive resistance in various crop species^2,10^. Despite the fundamental role of eIF4Es in protein translation in eukaryotic cells, loss-of-function mutations in these genes somehow result in distinct resistance spectra to plant viruses; eIF4Es-mediated recessive resistance is effective against potyviruses, bymoviruses, cucumoviruses, ipomoviruses, sobemoviruses, carmoviruses, and waikaviruses^13^. In *Arabidopsis thaliana*, selective involvement of eIF4Es is observed even in closely related viruses within the same genera, such as *Potyvirus* and *Polerovirus*^14–16^. Therefore, it is necessary to develop a recessive resistance cultivar with a virus resistance spectrum that is distinct from eIF4E-mediated resistance.

Several viruses belonging to the genus *Potexvirus* in the family *Alphaflexiviridae*, especially *Potato virus X* (PVX), *Cymbidium mosaic virus* (CymMV), *Pepino mosaic virus* (PepMV), and *Plantago asiatica mosaic virus* (PlAMV), infect economically important crops and cause devastating damage to their production^17–19^. Although several host susceptibility factors required for infection by some potexviruses have been identified^20–22^, to date, there has been no attempt to characterize resistance spectra based on susceptibility factor deficiencies across a range of viruses and plant species.

Previously, we successfully identified a novel susceptibility factor called *Essential for poteXvirus Accumulation 1* (*EXA1*), which is required for PlAMV infection^23^. *EXA1* has two functional domains: a GYF domain, comprising around 50 amino acids, which binds to proline-rich sequences^24^, and a Y-X_4_-L-L motif, predicted to bind to eIF4E^25^. EXA1 deficiency at the cellular level resulted in significantly reduced PlAMV RNA accumulation, suggesting that EXA1 is involved in PlAMV genome replication or translation^23^. Two other research groups have independently reported that *EXA1*/*MUSE11*/*PSIG1* is involved in plant immune responses against bacterial and oomycete pathogens, although its specific molecular function in plant immunity remains elusive^26,27^. Although these features suggest that *EXA1* is a promising target for breeding crops resistant to potexviruses, and possibly to other plant pathogens, it is unclear whether EXA1-mediated resistance is also effective against a range of potexviruses and their close relatives.

In the present study, we employed virus-induced gene silencing (VIGS) in *Nicotiana benthamiana* and *Solanum lycopersicum* to investigate the role of EXA1 in infection by a range of virus species. We further analyzed whether EXA1 homologs from multiple crops have functions that are complementary to endogenous EXA1 in *N*. *benthamiana*. Our results suggest that the function of EXA1 homolog(s) in multiple plant species is highly conserved in terms of its role in infection by a broad range of plant viruses, including potexviruses and other viruses in a closely related genus.

## Results

### Two *EXA1* homologous genes are identified in *N*. *benthamiana*

**Figure 1:**
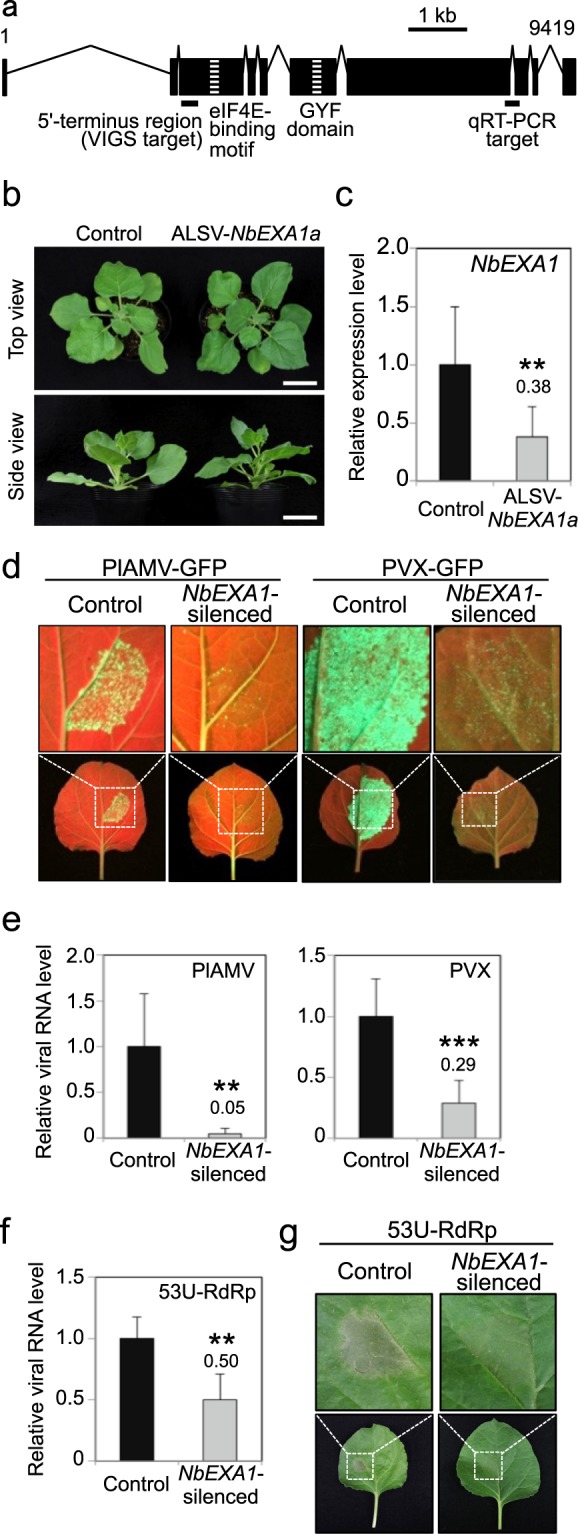
Cloning of *EXA1* homologs in *Nicotiana benthamiana* and their functional analysis in potexvirus infection. (**a**) Schematic image of the cDNA structure of *NbEXA1a*. GYF domain- and eIF4E-binding motif-encoding regions are depicted by stripes. Target regions for virus-induced gene silencing (VIGS) and quantitative reverse-transcription polymerase chain reaction (qRT-PCR) are indicated by bars under the image. (**b**) Morphological phenotypes of *NbEXA1*-silenced and control plants. Photographs were taken from the top (upper) and side (bottom) of plants at 27 days post-inoculation (dpi). Bars = 5 cm. (**c**) Relative accumulation of *NbEXA1* mRNA in *NbEXA1*-silenced and control plants. Total RNA was extracted at 27 dpi and analyzed using qRT-PCR. The mean level of *NbEXA1* transcript in control plants was used as the standard (1.0), and that in *NbEXA1*-silenced plants is shown above the bar. Error bars indicate standard deviation (SD) of 10 samples. **P < 0.01 (Student’s *t*-test). (**d**) GFP fluorescence emission of PlAMV-GFP- or PVX-GFP-inoculated leaves (bottom) of *NbEXA1*-silenced and control plants, with close-up views (upper). Inoculated leaves were observed under ultraviolet (UV) light at 6 and 4 dpi, respectively. (**e**) Accumulation of PlAMV-GFP and PVX-GFP genomic RNA in *NbEXA1*-silenced or control plants in Fig. 1d. Total RNA extracted from PlAMV-GFP- or PVX-GFP-inoculated leaves at 6 or 4 dpi, respectively, was analyzed using qRT-PCR. The mean level of viral RNA in control plants was used as the standard (1.0), and the level in *NbEXA1*-silenced plants is shown above the bar (also in Fig. 1f). Error bars represent SD of six (PlAMV-GFP) and 15 samples (PVX-GFP). ***P < 0.001 (Student’s *t*-test). (**f**) Accumulation of 53U-RdRp RNA in *NbEXA1-*silenced plants. Total RNA was extracted from inoculated areas expressing 53U-RdRp RNA at 4 dpi. The accumulation level of 53U-RdRp RNA was determined using qRT-PCR. Error bars indicate SD of four samples. (**g**) Symptoms of leaves (bottom) of *NbEXA1*-silenced and control plants inoculated with *Agrobacterium* carrying a plasmid expressing 53U-RdRp mRNA, with close-up views (upper). Photographs were taken at 4 dpi.

To identify *EXA1* homologs in *N*. *benthamiana*, we first performed a BLAST search (TBLASTN) using the *Arabidopsis* EXA1 (AtEXA1) amino acid sequence as a query against the *N*. *benthamiana* genome^28^. Two distinct predicted genes with sequences similar to those of *AtEXA1* were identified in two independent sequence contigs, Niben101Scf04831Ctg001 and Niben101Scf01371Ctg011. To confirm this result, we performed Southern blot analysis using two distinct probes within the predicted gene in Niben101Scf04831Ctg001: the 300-bp region in the 5′-terminus and the region corresponding to the GYF domain, which showed 98.3 and 97.7% identities, respectively, with the corresponding regions of another predicted gene in Niben101Scf01371Ctg011. Consistent with the results obtained by the BLAST search, these results also suggested that at least two genes hybridized with the probe in the *N*. *benthamiana* genome (Suppl. Fig. 1). Therefore, we named the *EXA1* homologs in Niben101Scf04831Ctg001 and Niben101Scf01371Ctg011 as *NbEXA1a* and *NbEXA1b*, respectively. Due to high nucleotide identity (92.8%) in the full-length genomic sequences, it was difficult to design specific polymerase chain reaction (PCR) primer sets to quantify the transcriptional level of each gene. To detect the transcripts of these genes, we tried to simultaneously amplify the 300-bp region in the 5′-termini of the two genes using a common primer set by reverse-transcription (RT)-PCR (Fig. 1a). Although over 50 clones obtained by RT-PCR and subsequently cloned PCR products were sequenced, all cDNA sequences matched the *NbEXA1a* sequence perfectly. This suggests that the transcription of *NbEXA1a* was readily detectable while that of *NbEXA1b* was undetectable in our experimental condition. We determined the genomic sequence of the full-length *NbEXA1a* gene and found substantial nucleotide changes compared with the existing sequence in *NbEXA1a*. Therefore, the newly determined *NbEXA1a* sequence was deposited in the DDBJ/EMBL/GenBank nucleotide database (accession no. LC171418). The NbEXA1a protein sequence is composed of 1,716 amino acids and shows 45.1% identity with AtEXA1. Consistent with AtEXA1^23^, a putative eIF4E-binding motif (Y-X_4_-L-L) and a GYF domain were found in exon 3 and exon 6 of *NbEXA1a*, respectively (Fig. 1a), indicating that NbEXA1a is also similar to AtEXA1 in its domain structure. In the following sections, we use the term *NbEXA1* to refer to both *NbEXA1a* and *NbEXA1b*.

### The NbEXA1 function related to viral infection is similar to that of AtEXA1

To silence *NbEXA1* expression, we employed a VIGS system based on apple latent spherical virus (ALSV)^29–31^. We cloned the 300-bp region in the 5′-terminus of *NbEXA1a* into the multi-cloning site of the ALSV vector. Using the 300-bp region of *NbEXA1a*, which has 98.3% identity with *NbEXA1b*, we expected that *NbEXA1b* expression would be knocked down along with *NbEXA1a* if *NbEXA1b* were authentically transcribed. We inoculated the constructed ALSV-*NbEXA1a* into the leaves of *N*. *benthamiana* to generate *NbEXA1*-silenced plants. As a negative control, we used *N*. *benthamiana* inoculated with empty ALSV vector (control plants). At 27 days post-inoculation (dpi), no obvious phenotypic differences were observed in the ALSV-*NbEXA1a*-inoculated plants (Fig. 1b). Total RNA was extracted from the uninoculated upper leaves at 27 dpi, and *NbEXA1* expression was analyzed by quantitative RT (qRT)-PCR using *NbEXA1*-specific primers and *N*. *benthaminana 18S* rRNA (*Nb18S*) gene-specific primers as an internal control. The accumulation level of *NbEXA1* mRNA was significantly lower in ALSV-*NbEXA1a*-infiltrated plants compared with control plants (Fig. 1c).

To test whether NbEXA1 is required for viral infection in *N*. *benthamiana*, we inoculated the upper leaves of *NbEXA1*-silenced and control plants with two potexviruses, PlAMV and PVX, both of which had been tagged with green fluorescent protein (GFP). GFP fluorescence of PlAMV-GFP or PVX-GFP, which indicates viral accumulation, was clearly observed in the inoculated leaves of control plants at 6 or 4 dpi, respectively. In contrast, GFP fluorescence of PlAMV-GFP or PVX-GFP decreased drastically in inoculated leaves of *NbEXA1*-silenced plants compared with control plants (Fig. 1d). To quantify the accumulation levels of the viral RNAs, we measured viral RNA accumulation in the virus-inoculated areas using qRT-PCR. The accumulation of PlAMV-GFP and PVX-GFP was significantly lower in *NbEXA1*-silenced plants than in control plants (Fig. 1e), suggesting that NbEXA1 is required for efficient accumulation of PlAMV and PVX in *N*. *benthamiana*. To exclude the possibility that *NbEXA1* silencing influences *Agrobacterium* infection, we mechanically inoculated these viruses into *NbEXA1*-silenced and control plants using sap prepared from PlAMV-GFP-infected *N*. *benthamiana*. Consistent with the above results, GFP signal intensity and viral accumulation were significantly lower in *NbEXA1*-silenced plants than in control plants (Suppl. Fig. 2), indicating that the decrease in viral RNA accumulation shown in Fig. 1e was not due to interference with *Agrobacterium* infection by *NbEXA1* knockdown, but rather to the involvement of NbEXA1 in viral accumulation. Together, these results indicate that NbEXA1 is required for efficient viral accumulation of the potexviruses PlAMV and PVX in *N*. *benthamiana*.

Despite the apparent requirement of NbEXA1 for viral accumulation, it remained unclear whether NbEXA1 is involved in viral propagation within the initially infected cells or in cell-to-cell movement to adjacent healthy cells. We previously reported that AtEXA1 is involved in the cellular accumulation of the virus because a PlAMV-derived replicon, referred to as 53U-RdRp, encoding an RNA-dependent RNA polymerase (RdRp) combined with the 5′- and the 3′-terminal regions of the PlAMV genome, was inhibited in *Arabidopsis exa1-1* mutants^23^. To investigate whether NbEXA1 is involved in viral propagation in the initially infected cells, *Agrobacterium* carrying a plasmid expressing the 53U-RdRp replicon was inoculated into the leaves of *NbEXA1*-silenced and control plants. To quantify the accumulation of 53U-RdRp, we extracted total RNA from the inoculated leaves of the *NbEXA1*-silenced or control plants and analyzed its levels using qRT-PCR. The accumulation of 53U-RdRp in the *NbEXA1*-silenced plants was significantly lower than in control plants (Fig. 1f). 53U-RdRp induces necrotic symptoms in *N*. *benthamiana* due to high levels of RdRp accumulation, which may be translated from the replicated mRNA as well as from the initially transcribed mRNA^32^. Consistently, 53U-RdRp caused necrotic symptoms at 4 dpi in control plants, but not in *NbEXA1*-silenced plants (Fig. 1g). These results suggest that NbEXA1, like AtEXA1, is involved in the propagation of PlAMV in the initially infected cells.

### NbEXA1 is required for infection by several viral species of two distinct genera

**Figure 2:**
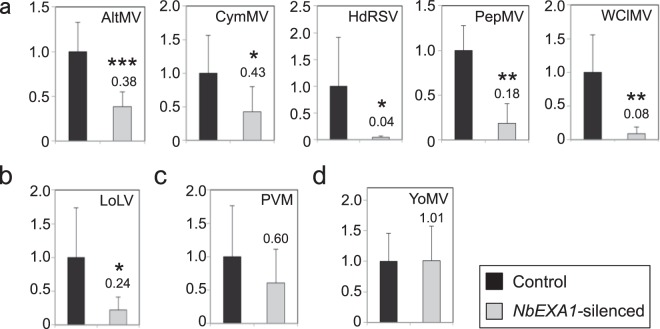
Evaluation of the accumulation of viral RNA in *NbEXA1*-silenced or control plants using qRT-PCR. Plants were mechanically inoculated with (**a**) potexviruses, (**b**) a lolavirus, (**c**) a carlavirus, and (**d**) a tobamovirus. Total RNA was extracted from inoculated leaves at 4 dpi for AltMV, CymMV, HdRSV, PepMV, PVM, and YoMV; 5 dpi for LoLV; and 8 dpi for WClMV. The mean level of viral RNA in control plants was used as the standard (1.0), and that in *NbEXA1*-silenced plants is shown above the bars. Error bars represent SD of eight samples, except for PepMV (four samples). *P < 0.05; **P < 0.01; and ***P < 0.001 (Student’s *t*-test).

The essential function of NbEXA1 in the accumulation of two potexviruses led us to investigate whether NbEXA1 is also involved in the accumulation of other viruses in the same genus or closely related genera. We focused on eight distinct virus species known to infect *N*. *benthamiana*, from four viral genera in three families. In the family *Alphaflexiviridae*, AltMV, CymMV, *Hydrangea ringspot virus* (HdRSV), PepMV, and *White clover mosaic virus* (WClMV) were selected from the genus *Potexvirus*, and LoLV was selected from the genus *Lolavirus*. In the family *Betaflexiviridae*, which is closely related to *Alphaflexiviridae*, *Potato virus M* (PVM) was selected from the genus *Carlavirus*. We also selected *Youcai mosaic virus* (YoMV) from the genus *Tobamovirus* in the family *Virgaviridae*, which is distantly related to the above two families. These viruses were inoculated into the leaves of *NbEXA1*-silenced and control plants, and viral accumulation in the inoculated leaves was measured via qRT-PCR with primers specific to each virus. The accumulation of AltMV was significantly lower in *NbEXA1*-silenced plants than in control plants (Fig. 2a). In contrast, the accumulation of YoMV did not differ significantly between *NbEXA1*-silenced plants and control plants (Fig. 2d), indicating that NbEXA1 functions equivalently to AtEXA1 in AltMV and YoMV infection^23^. Moreover, the accumulation of four potexviruses (CymMV, HdRSV, PepMV, and WClMV) in *NbEXA1*-silenced plants was significantly lower than in control plants (Fig. 2a). Remarkably, LoLV accumulation was also significantly compromised in *NbEXA1*-silenced plants (Fig. 2b). Lower levels of PVM accumulation were repeatedly observed in *NbEXA1*-silenced plants than in controls, although these differences were not statistically significant (Fig. 2c). These results demonstrate that NbEXA1 was also required for propagation of the tested viruses in the *Potexvirus* and *Lolavirus* genera in *N*. *benthamiana*.

### NbEXA1 function related to viral infection is complemented by EXA1 homologs of tomato and rice

**Figure 3:**
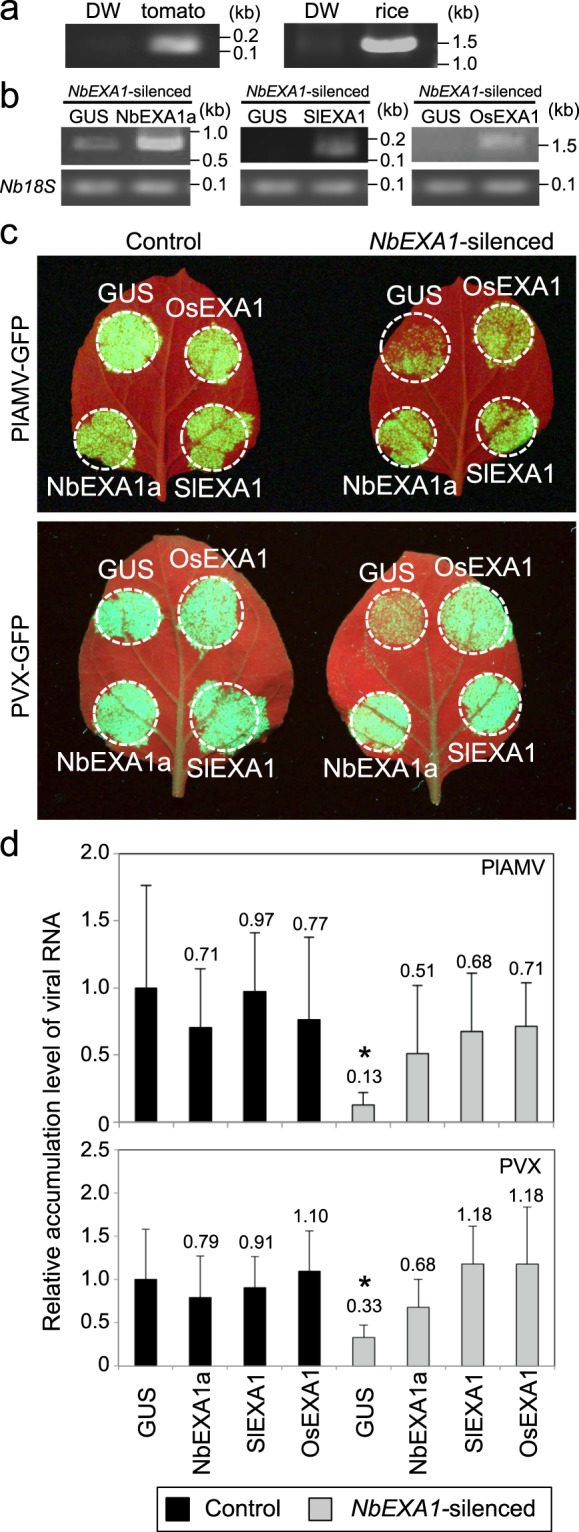
EXA1 homologs in tomato and rice complement the role of EXA1 in infection by PlAMV and PVX. (**a**) Expression analysis of *SlEXA1* and *OsEXA1* using RT-PCR in tomato and rice plants. Total RNA was extracted from tomato and rice leaves. DW: distilled water. (**b**) The accumulation of *NbEXA1a*, *SlEXA1*, and *OsEXA1* transcripts in *N*. *benthamiana* leaves inoculated with *Agrobacterium* carrying a plasmid encoding the genomic sequence of each *EXA1* homolog and *GUS*. *Nb18S* was used as the internal control. (**c**) Functional complementation analysis of PlAMV-GFP (upper) and PVX-GFP (bottom) infection by the transient expression of *NbEXA1a*, *SlEXA1*, or *OsEXA1* in *NbEXA1*-silenced (right) and control (left) plants. Accumulation of PlAMV-GFP or PVX-GFP co-expressed with each *EXA1* homolog and GUS was observed under UV light at 4 dpi. (**d**) Quantification of the accumulation of PlAMV-GFP and PVX-GFP RNA in *NbEXA1*-silenced and control plants using qRT-PCR. Total RNA extracted from the inoculated leaves at 4 dpi. Error bars represent SD of six (PlAMV-GFP) and three (PVX-GFP) samples. The mean level of viral RNA in control plants expressed with GUS was used as the standard (1.0), and scores for other conditions are shown above the bars. *P < 0.05. Analysis of variance (ANOVA) with Dunnett’s multiple comparison was used to test for significant differences among means compared to the GUS-expressing area in the control plants.

We next sought to determine whether the function of NbEXA1 in viral infection is conserved among *EXA1* homologs from other plant species. Although *EXA1* homologs were predicted in dicot and monocot plants (tomato and rice) in our previous study^23^, their biological functions in viral infection remain unknown. Predicted protein sequences of *EXA1*-homologous genes in tomato (*SlEXA1*) and rice (*OsEXA1*) showed 79.2 and 33.8% identity, respectively, with *NbEXA1a* (Table 1). To confirm the transcription of *SlEXA1* and *OsEXA1*, we performed RT-PCR to detect *SlEXA1* and *OsEXA1* mRNAs transcribed endogenously in the original plants. The specific primers were designed based on the nucleotide sequences of *SlEXA1* and *OsEXA1* genes obtained from the Phytozome database^33^. PCR products of the expected size were obtained from each plant species (Fig. 3a), demonstrating that these genes are transcriptionally expressed in tomato and rice.

**Table 1:** Amino acid sequence identities of EXA1 homologs (%).

	AtEXA1	NbEXA1a	SlEXA1
NbEXA1a	45.1		
SlEXA1	48.9	79.2	
OsEXA1	35.5	33.8	37.5

To determine whether these homologs have the same function as NbEXA1 in viral infection, we performed a transient complementation assay combined with a VIGS-based *NbEXA1* knockdown technique in *N*. *benthamiana*. In this assay, we co-expressed one of the putative EXA1 homologs along with PlAMV-GFP or PVX-GFP into leaves of *NbEXA1*-silenced or control non-silenced plants. Transient expression of each putative *EXA1* transcript in *N*. *benthamiana* leaves was confirmed by RT-PCR (Fig. 3b). In control plants, GFP fluorescence of PlAMV-GFP or PVX-GFP was observed in the EXA1 homolog-expressing areas at levels comparable to those of GUS-expressing areas. In *NbEXA1*-silenced plants, although only weak GFP fluorescence was observed in GUS-expressing areas, intense GFP fluorescence was clearly observed in areas where NbEXA1a was expressed, indicating the successful complementation by the transient expression of NbEXA1a. Consistent with this finding, GFP fluorescence of PlAMV-GFP or PVX-GFP was recovered in areas where SlEXA1 and OsEXA1 were expressed (Fig. 3c). To analyze these results in detail, we measured viral accumulation levels in the infiltrated areas using qRT-PCR. In non-silenced control plants, the expression of the putative EXA1 homologs showed no significant impact on viral accumulation compared with GUS controls. In *NbEXA1*-silenced plants, viral accumulation was recovered by expression of the putative EXA1 homologs as well as NbEXA1a compared with the GUS control (Fig. 3d). These results indicate that SlEXA1 and OsEXA1, EXA1 homologs from tomato and rice, respectively, as well as NbEXA1a may support potexviral infection in *N*. *benthamiana*.

### Silencing of *EXA1* homolog compromises PepMV RNA accumulation in tomato

**Figure 4:**
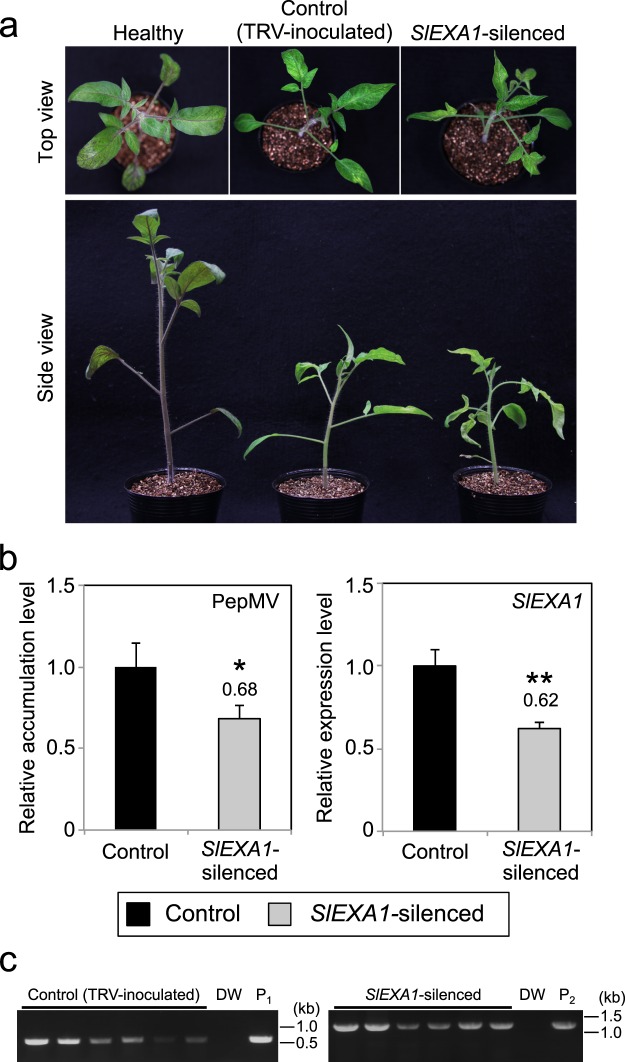
Downregulation of *SlEXA1* expression inhibits PepMV RNA accumulation in tomato. (**a**) Morphological phenotypes of control and *SlEXA1*-silenced plants. Tomato seedlings were inoculated with TRV (control) and TRV-*SlEXA1* (*SlEXA1*-silenced) by agroinfiltration. Photographs were taken from the top (upper) and side (bottom) of plants at 4 weeks post-inoculation. Un-inoculated healthy plants served as visual controls. (**b**) Quantification of the accumulation of PepMV RNA and *SlEXA1* mRNA in control and *SlEXA1*-silenced plants using qRT-PCR. Six control plants and six *SlEXA1*-silenced plants were mechanically inoculated with PepMV. Total RNA extracted from PepMV-inoculated leaves at 5 dpi served as templates for qRT-PCR analysis of PepMV RNA and *SlEXA1* mRNA. Error bars represent the standard error (SE) of six samples. The mean level of viral RNA or *SlEXA1* mRNA in control plants was used as the standard (1.0), and scores for other conditions are shown above the bars. *P < 0.05; **P < 0.01 (Student’s *t*-test). (**c**) Confirmation of TRV and TRV-*SlEXA1* infection by RT-PCR. These RNA samples were the same as in (**b**). P_1_, pTRV2; P_2_, pTRV2-*SlEXA1*.

Functional complementation of endogenous NbEXA1 by EXA1 homologs from tomato and rice led us to postulate that EXA1 is also a host susceptibility factor for potexviruses when infecting their original host plants, especially crops. To test this hypothesis, we next attempted to silence the expression of *SlEXA1* (*Solyc04g080240*.*2*), an *EXA1* homolog in tomato, employing a TRV-mediated VIGS system^34,35^. A TRV VIGS vector carrying the *SlEXA1* fragment TRV-*SlEXA1* was pre-inoculated into the cotyledons of 1-week-old tomato plants via an agro-infiltration method. At 4 weeks after inoculation, both TRV- and TRV-*SlEXA1*-inoculated plants showed mosaic symptoms and stunting compared with un-inoculated plants, with no obvious visual differences between the two (Fig. 4a). After observing this phenotype, the upper leaves were mechanically inoculated with PepMV. At 5 dpi, total RNA was extracted from inoculated leaves, and we verified the infection of pre-inoculated TRV in both TRV- and TRV-*SlEXA1*-inoculated plants by RT-PCR (Fig. 4c). We then analyzed PepMV accumulation and *SlEXA1* expression by qRT-PCR. Compared with TRV-inoculated control plants, significantly lower levels of viral RNA accumulation and *SlEXA1* expression were observed in TRV-*SlEXA1*-inoculated plants (Fig. 4b). This result suggests that SlEXA1 is involved in PepMV infection in tomato, as with NbEXA1.

### *EXA1* homologs are encoded in a wide range of plant species

**Figure 5:**
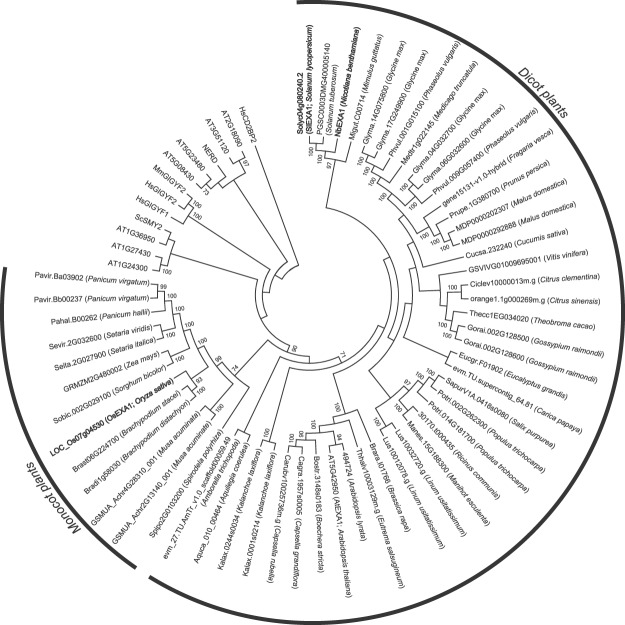
Phylogenetic tree constructed using the neighbor-joining method with full-length amino acid sequences of all EXA1 homologs. *Homo sapiens* HsCD2BP2 was used as an outgroup. Protein sequences used in this analysis are listed in Suppl. Table 2. Numbers at the nodes represent bootstrap values greater than 70% obtained from 1,000 replicates. EXA1 homologs written in bold type were analyzed in this study.

To investigate whether *EXA1* homologs are conserved across a wide range of plant species, we performed a BLAST search against complete and draft genome sequences deposited in the Phytozome database using the NbEXA1a amino acid sequence as a query. We obtained 51 sequences containing a GYF domain and an eIF4E-binding motif from among 39 plant species (Suppl. Fig. 3). We obtained exactly the same set of sequences using the AtEXA1 amino acid sequence as a query. Importantly, these sequences were found in the genomes of a wide range of plant species, including vegetables, flowers, and fruits, and showed 33.9–78.3% or 32.6–92.7% identity with NbEXA1a or AtEXA1, respectively. To examine the phylogenetic relationships among *EXA1* homologs, we built a phylogenetic tree using the obtained full-length amino acid sequences (Fig. 5). *EXA1* homologs in dicot plants were clustered in one monophyletic group along with NbEXA1a and AtEXA1, and another distinct cluster was found among monocot plants. This phylogenetic relationship among EXA1 homologs supported the hypothesis that EXA1 homologs arose from an EXA1-like gene in a common ancestor of dicots and monocots. All *EXA1* homologs in dicot and monocot plants were located in a distinct group compared with the other GYF domain-containing proteins, supporting the notion that all of the retrieved sequences are *EXA1* homologs. Including the GYF domain and eIF4E-binding motif, the sequence comparison among all *EXA1* homologous genes revealed that several amino acid sequence stretches were found among nearly all of the genes (Suppl. Fig. 4), suggesting that uncharacterized functional domains or motifs might be related to their common functions. Most plants encode a single *EXA1* homolog in their genome, whereas some plants (e.g., banana, soybean, and *N*. *benthamiana*) encode two or four *EXA1* homologs. This phenomenon is thought to be due to whole-genome duplication in these lineages; the same can be expected for other allotetraploid species^28,36,37^.

## Discussion

In the present study, to examine whether EXA1 originally isolated from the model plant *A*. *thaliana* is a promising target for virus resistance breeding, we addressed two important questions: which viruses require EXA1 to achieve host infection, and whether the function of EXA1 homologs is conserved among plant species. To address these questions, we analyzed the accumulation of various viruses in *NbEXA1*-silenced *N*. *benthamiana*. The accumulation of seven potexviruses and one lolavirus that is closely related to potexviruses significantly decreased in *NbEXA1*-silenced plants (Figs 1, 2), demonstrating that NbEXA1 is required for these viruses to accumulate in *N*. *benthamiana*. Because all of these viruses belong to *Alphaflexiviridae*, NbEXA1 may be commonly involved in the infection cycles of members of this family. In contrast, the accumulation of YoMV (genus: *Tobamovirus*; family: *Virgaviridae*) did not differ between *NbEXA1*-silenced and control plants. In *A*. *thaliana*, systemic infection of YoMV, *Turnip crinkle virus* (genus: *Carmovirus*; family: *Tombusviridae*), and *Turnip yellow mosaic virus* (genus: *Tymovirus*; family: *Tymoviridae*) was not altered in the *exa1-1* mutant^23^. These results suggest that EXA1 is unnecessary for infection by viruses unrelated to *Alphaflexiviridae*. However, it should be noted that the accumulation of PVM in the family *Betaflexiviridae*, which is classified into the same viral order (*Tymovirales*) as *Alphaflexiviridae*, tends to decrease in *NbEXA1*-silenced plants. Thus, we speculate that NbEXA1 may also be partially involved in viral accumulation in *Betaflexiviridae*.

Based on our previous and current studies using the PlAMV replicon 53U-RdRp, we demonstrated that the early infection step of PlAMV was significantly impaired by EXA1 deficiency in two host plants^23^. Moreover, EXA1 deficiency did not influence the infection capability of several viruses distantly related to *Alphaflexiviridae*. Although the molecular function of EXA1 in viral infection remains unclear, it is likely that EXA1 functions as a relevant susceptibility factor specifically recruited for the initial infection steps of alphaflexiviruses. Recently, two groups independently reported the function of EXA1/MUSE11/PSIG1 in plant innate immunity^26,27^. Interestingly, based on several lines of evidence, Wu *et al*. claimed that EXA1/MUSE11 negatively regulates plant innate immunity against bacterial and oomycete pathogens, possibly through translational repression of immune receptors^27^. This claim is supported in part by the fact that EXA1/PSIG1 localizes to processing bodies^26^, distinct cytoplasmic foci containing factors involved in translational repression and the mRNA decay machinery^38^. It remains possible that EXA1 negatively regulates the translation of an uncharacterized immune receptor that specifically recognizes alphaflexiviruses. Further analysis is essential to reveal the molecular function of EXA1 in infection by alpha- and betaflexiviruses.

To analyze the functional consistency of EXA1 homologs in viral infection, we performed complementation analyses in *NbEXA1*-silenced plants by transiently expressing EXA1 homologs from tomato and rice as well as NbEXA1a. When NbEXA1 was transiently expressed along with viral RNA in *NbEXA1*-silenced plants, viral accumulation levels became similar to those in GUS- and NbEXA1a-co-expressing areas of control plants (Fig. 3). These results suggest that the function of the EXA1 homolog in viral infection is also conserved in the solanaceous plant *N*. *benthamiana*. Similarly, transient expression of EXA1 homologs from tomato and rice supported PlAMV and PVX accumulation in *NbEXA1*-silenced plants, indicating that these homologs are functionally equivalent to NbEXA1 in viral accumulation (Fig. 3). Additionally, downregulation of *SlEXA1*, an *EXA1* homolog of tomato, did result in lower PepMV RNA accumulation in tomato (Fig. 4). From these results, we conclude that EXA1 homologs in diverse plant species are necessary susceptibility factors for potexvirus infection. Because *EXA1* homologous genes are conserved across various plant species (Fig. 5), they may represent a promising target for potexvirus resistance breeding in crops.

Most plants encode a single *EXA1* homolog gene in their genomes, whereas some plants, such as *N*. *benthamiana*, soybean, and banana, encode multiple homologous genes. The multiplicity of EXA1 homologs may be attributed to the occurrence of whole-genome duplication or genome polyploidization in a plant lineage^28,36,37^. In the present study, we found that *N*. *benthamiana* encodes at least two *EXA1*-homologous sequences and confirmed that mRNA is transcribed mainly from only one of these, which we named *NbEXA1a* (Fig. 1, Suppl. Fig. 1). These results suggest that *NbEXA1b* may be a pseudogene in *N*. *benthamiana*. However, it is also possible that EXA1 homologs function in a redundant manner. Among the well-characterized recessive resistance genes in *A*. *thaliana*, there are two *eIFiso4G* genes: *eIFiso4G1* and *eIFiso4G2*. Although a single mutant of each gene does not compromise the infectivity of *Turnip mosaic virus* (TuMV, genus: *Potyvirus*), simultaneous mutation of both homolog genes does impair TuMV infection, indicating that eIFiso4G1 and eIFiso4G2 have redundant functions in TuMV infection^14^. Therefore, in plants encoding multiple *EXA1* homologs, it is also conceivable that the homologous genes have redundant functions in viral infectivity.

One of the most important points to consider in the development of virus-resistant plant cultivars is the possibility of unexpected harmful effects on crop production by loss-of-function mutations or downregulation via RNA silencing. The growth rate of the *exa1-1* mutant, which has a null mutation in *AtEXA1*, is slightly slower than that of wild-type *Arabidopsis*^23^. Null mutation of EXA1 homologs in other plant species might delay plant growth or affect crop production. Ouibrahim *et al*. demonstrated that recessive resistance to *Watermelon mosaic virus* (WMV, genus: *Potyvirus*) in the *Arabidopsis* Cvi-0 ecotype is due to a single amino acid substitution in the *cPGK2* gene^39^. However, null mutation of *cPGK2* results in a seedling-lethal phenotype, suggesting that cPGK2 is essential for *Arabidopsis* growth, and the substituted amino acid residue in the *cPGK2* gene of the Cvi-0 ecotype is crucial for molecular interaction of cPGK2 with WMV^39^. Further extensive studies to reveal the molecular function of EXA1 in potexviral infection are necessary to identify potential target sites in *EXA1* homologs critical for potexviral infection, but not for normal plant growth. In *A*. *thaliana*, resistance pyramiding strategy has recently been shown to be effective against several potyviruses including resistance-breaking isolates of TuMV and a polerovirus, in which a lethal phenotype of the *eif4e1eifiso4e* double-knockout mutant was rescued by introducing a synthetic *eIF4E1* allele with multiple amino acid changes associated with potyvirus resistance in naturally occurring *Pisum sativum* alleles^40^. Given that *EXA1* homologs are encoded by various plant species including tomato and rice, such an elegant strategy should be adopted to develop EXA1-tailored potexvirus-resistant cultivars in several commercially important crops with durable and broad-spectrum resistance, before the advent of an EXA1-mediated resistance-breaking virus.

## Methods

### Plant materials and growth conditions

*N*. *benthamiana*, tomato (*S*. *lycopersicum* cultivar Micro-Tom), and rice (*Oryza sativa* cultivar Koshihikari) were grown in growth chambers under 16-h light/8-h dark conditions at 25 °C, except for a VIGS assay of tomato (*S*. *lycopersicum* cultivar Brandywine black), which was grown at 22 °C, which is the optimal temperature for silencing in tomato^34^.

### BLAST search

To retrieve *EXA1* homolog sequences, a TBLASTN search was performed using the AtEXA1 protein sequence as a query against the Sol Genomic Network (https://solgenomics.net/) for *N*. *benthamiana* and the Phytozome database v. 11 (http://phytozome.jgi.doe.gov/pz/portal.html) for the other plant species.

### DNA extraction

For DNA extraction, 0.3 g of plant leaf was homogenized in liquid nitrogen and mixed with 700 µL 2× CTAB buffer (100 mM Tris-HCl [pH 8.0], 1.4 M NaCl, 20 mM EDTA, 2% CTAB, and 0.2% 3-mercapto-1,2-propanediol), and incubated at 65 °C for 30 min. After centrifugation, 400 µL chloroform/isoamyl alcohol (24:1, v/v) was added to the supernatant, mixed, and centrifuged. The supernatant was mixed with 5 ng RNase A (Nippon Gene, Tokyo, Japan) and incubated at 37 °C for 1 h. After incubation, 400 µL chloroform/isoamyl alcohol was added, mixed, and centrifuged. The supernatant was then subjected to isopropanol precipitation.

### Southern blot analysis

Total genomic DNA (5 µg) of *N*. *benthamiana* was digested with *Dra* I (Nippon Gene), *Hin*d III-HF, or *Nde* I restriction enzymes (New England BioLabs, Ipswich, MA, USA). Southern blot hybridization was performed using the DIG application kit (Roche, Basal, Switzerland) according to the manufacturer’s instructions. Specific DNA probes for the detection of *NbEXA1* were synthesized using the PCR DIG Probe Synthesis Kit (Roche) according to the manufacturer’s protocols using the primer sets *Xh*-*NbEXA1*-268F/*Bm*-*NbEXA1*-567R or *NbEXA1*g-5015F/*NbEXA1*g-5500R, and with *N*. *benthamiana* genomic DNA as a template.

### RNA isolation, RT-PCR, and qRT-PCR

Total RNA was extracted from the plants using ISOGEN (Nippon Gene) and treated with DNase I (Takara Bio, Shiga, Japan), or using the ISOSPIN Plant RNA Kit (Nippongene) following the manufacturer’s instructions. cDNA was synthesized using a High-Capacity cDNA Reverse Transcription Kit (Thermo Fisher Scientific, Waltham, MA, USA). RT-PCR was conducted using KOD FX (Toyobo, Osaka, Japan) according to the manufacturer’s protocol. To detect the mRNA of *NbEXA1*, *SlEXA1*, and *OsEXA1*, RT-PCR was performed using the primer sets *NbEXA1*-rt12F/*Nt*-*NbEXA1*-5451R, *Kp*-*SlEXA1*-5U-F/*SlEXA1*g-3301R, and *Bm*-*OsEXA1*-F/*OsEXA1*-1480R, respectively. TRV and TRV-*SlEXA1* detection was performed using the SuperScript III One-Step RT-PCR System (Invitrogen, Carlsbad, CA, USA) according to the manufacturer’s instructions, using the primer pair TRV-F/TRV-R. qRT-PCR assays were performed using the Thermal Cycler Dice Real Time System and SYBR Premix Ex Taq II (Takara Bio). The *Nb18S* rRNA gene and *SlActin* were used as internal standards for qRT-PCR analysis in *N*. *benthamiana* and *S*. *lycopersicum*, respectively. All primers used in this study are listed in Suppl. Table 1. Primers for the quantification of the viral RNA are all targeted for plus strand of each viral genome. Of these, primers for AltMV, CymMV, HdRSV, LoLV, PlAMV, YoMV are targeted for RdRp coding region, while primers for PepMV, PVM, PVX, WClMV are targeted for coat protein coding region.

### Plasmid construction

To silence *NbEXA1* expression, we used the ALSV VIGS vectors pCAM-ALSV1 and pCAM-ALSV2^29–31^, and constructed pCAM-ALSV2-*NbEXA1a*. The 300-bp N-terminal fragment of *NbEXA1a* (Fig. 1a) was amplified from total *N*. *benthamiana* DNA using the primers *Xh*-*NbEXA1*-268F and *Bm*-*NbEXA1*-567R, and inserted into pCAM-ALSV2 between the *Xho* I and *Bam*H I sites.

Downregulation of *SlEXA1* expression was achieved using pTRV2-*SlEXA1*, which was derived from the TRV VIGS vectors pTRV1 and pTRV2^35^. The partial 501-bp fragment of *SlEXA1* was amplified from cDNA synthesized from total *S*. *lycopersicum* RNA using the primers *Xh*-*SlEXA1*-865F and *Bm*-*SlEXA1*-1365R, and inserted into pTRV2 between the *Xho* I and *Bam*H I sites.

Viral clones were constructed as follows. The full-length cDNA sequences of AltMV^41^, CymMV^42^, HdRSV^43^, and LoLV^44^ were amplified using the primer sets AltMV-1F/KpGR3nest, CymMV-1F/KpGR3nest, HdRSV-24F/KpGR3nest, and Lol-1F/Lol-7650R-polyA40, respectively. DNA fragments harboring the 35 S promoter from pCAMBIA1301 and the 5′-terminal sequence of each virus genome were amplified from pPPVOu binary vectors^45^ using the primer sets KpGR3nesF/Alt35SR, KpGR3nesF/CymMV_35S_R, and KpGR3nesF/Hd35SR. DNA fragments harboring NOS terminators from pCAMBIA1301 were amplified along with polyA using the primer set 20polyA-NOS/Lol35SR. Full-length viral cDNAs of AltMV, CymMV, HdRSV, and LoLV were then assembled with the PCR products of the 35S promoter and NOS terminator into the pCAMBIA1301 vector using a GeneArt Seamless Cloning and Assembly kit (Thermo Fisher Scientific) to generate pAltMV, pCymMV, pHdRSV, and pLoLV, respectively. A DNA fragment harboring the 35S promoter and full-length cDNA from WClMV-RC^46^ was subcloned into the *Eco*R I and *Pma*C I sites of pCAMBIA1301 (pWClMV).

Genomic sequences of *NbEXA1a*, *SlEXA1*, and *OsEXA1* were amplified by PCR from total DNA of *N*. *benthamiana*, tomato, and rice using the primer sets *NbEXA1*-F/*NbEXA1*-R, *Kp*-*SlEXA1*-5U-F/*Nt*-*SlEXA1*-R, and *Bm*-*OsEXA1*-F/*Nt*-*OsEXA1*-R, respectively. The PCR products of *NbEXA1a* and *SlEXA1* were inserted into the *Kpn* I and *Not* I sites of Gateway Entry vector pENTA^47^ and the products of *OsEXA1* were inserted into the *Bam*H I and *Not* I sites of pENTA. Each insert was cloned into the pEarleyGate100 vector^48^ using Gateway LR Clonase II Enzyme Mix (Thermo Fisher Scientific), resulting in p35S-*NbEXA1a*g, p35S-*SlEXA1*g, and p35S-*OsEXA1*g, respectively.

#### Agrobacterium inoculation

For *Agrobacterium* inoculation, pCAM-ALSV1, pCAM-ALSV2, pCAM-ALSV2-*NbEXA1a*, pTRV1, pTRV2, pTRV2-*SlEXA1*, pBin-P19 (a binary vector containing the sequence of tomato bushy stunt virus p19), pAltMV, pCymMV, pHdRSV, pLoLV, pWClMV, pLi1-sGFP^49^ (an infectious cDNA clone of PlAMV-GFP), p53U-RdRp^32^, pPVX-GFP^50^, p35S-*NbEXA1*g, p35S-*SlEXA1*g, and p35S-*OsEXA1*g were used to transform *A*. *tumefaciens* strain EHA105. These *Agrobacterium* cells were inoculated as previously described^51^. GFP fluorescence was observed under ultraviolet (UV) light at 365 nm (LED365-9UV033B; OptoCode, Tokyo, Japan) or 470 nm (LED470/L-HNDY; OptoCode) and photographed using a Canon EOS Kiss IV digital camera with a yellow filter (Y2; Kenko, Tokyo, Japan) or a UV-absorbing filter (SC-52; Fujifilm, Tokyo, Japan). Obtained images were processed using the Adobe Photoshop CS4 software.

To induce *NbEXA1* silencing, expanded leaves of 3-week-old *N*. *benthamiana* plants were inoculated with a mixture of three *Agrobacterium* cultures containing pCAM-ALSV1, pCAM-ALSV2-*NbEXA1a*, and pBin-P19. Their final optical density at 600 nm (OD_600_) was adjusted to 0.5 as previously described^52^.

To downregulate *SlEXA1* expression, cotyledons of 1-week-old *S*. *lycopersicum* (cultivar Brandywine black) plants were inoculated with a mixture of *Agrobacterium* cultures containing pTRV1 and pTRV2-*SlEXA1*. Their final optical density at OD_600_ was adjusted to 1.0 before mixing at a ratio of 1:1.

### Mechanical inoculation

*N*. *benthamiana* was mechanically inoculated with sap from PlAMV-GFP-infected *N*. *benthamiana* leaves in 0.1 M phosphate buffer, pH 7.0, as previously described^52^. PepMV (PV-0973; Leibniz-Institut DSMZ), PVM (MAFF307027; National Agriculture and Food Research Organization GeneBank), and YoMV^53^ were mechanically inoculated using the same procedure. *S*. *lycopersicum* was also mechanically inoculated as described above.

### Alignment and phylogenetic analysis of EXA1 homologs

Alignment and phylogenetic analysis were performed using the GeneDoc 2.6 and MEGA6.06 software, respectively, based on a multiple alignment created using the MUSCLE software (http://www.ebi.ac.uk/Tools/msa/muscle/).

## Supplementary information

Suppl. Figures

Suppl. Table 2
