# Supplementary material for: Functional conservation of EXA1 among diverse plant species for the infection by a family of plant viruses
Source: Sci Rep. 2019 Apr 11;9:5958. doi: 10.1038/s41598-019-42400-w (PMC6459814; doi:10.1038/s41598-019-42400-w)
Supplement: Supplementary file 1 — Suppl. Figures [file 41598_2019_42400_MOESM1_ESM.pdf]

Supplementary information

Functional conservation of EXA1 among diverse plant species for the infection by a family of plant viruses

Akira Yusa†, Yutaro Neriya†, Masayoshi Hashimoto, Tetsuya Yoshida, Yuji Fujimoto, Naoi Hosoe, Takuya Keima, Kai Tokumaru, Kensaku Maejima, Osamu Netsu, Yasuyuki Yamaji, and Shigetou Namba*

†These authors contributed equally to this work.

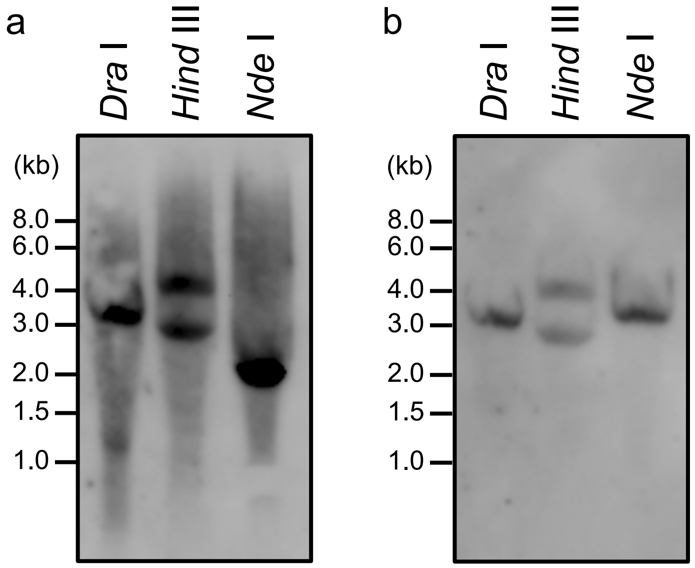

Supplementary Figure 1. Southern blot analysis performed to detect *EXA1* homologs in *N. benthamiana*

(a) the N-terminal 300-bp region and (b) GYF domain-specific DNA probes were used respectively. The restriction enzymes used to digest total DNA are shown above the images. Marker positions are indicated to the left of the images.

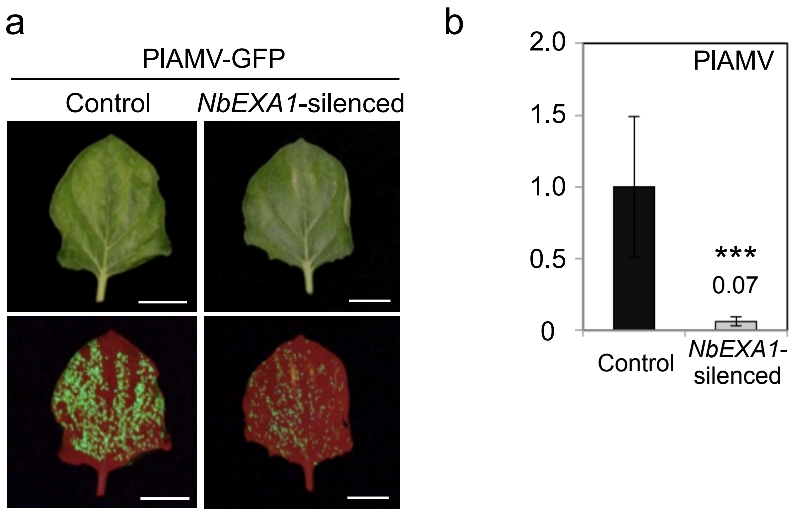

Supplementary Figure 2. Accumulation of mechanically inoculated PlAMV-GFP decreased in *NbEXA1*-silenced plants compared to control plants.
(a) *NbEXA1*-silenced and control plants were mechanically inoculated with PlAMV-GFP. Green fluorescent protein (GFP) fluorescence emission from PlAMV-GFP was visualized under ultraviolet (UV) light at 4 days post-inoculation (dpi). (b) Accumulation of mechanically inoculated PlAMV-GFP in *NbEXA1*-silenced and control plants. Total RNA extracted from inoculated leaves was analyzed by quantitative reverse-transcription polymerase chain reaction (qRT-PCR) at 4 dpi. The mean level of viral RNA in control plants was used as the standard (1.0), and that in *NbEXA1*-silenced plants is shown above the bar. Error bars represent standard deviation (SD) of eight samples. *** $P < 0.001$ (Student's *t*-test).

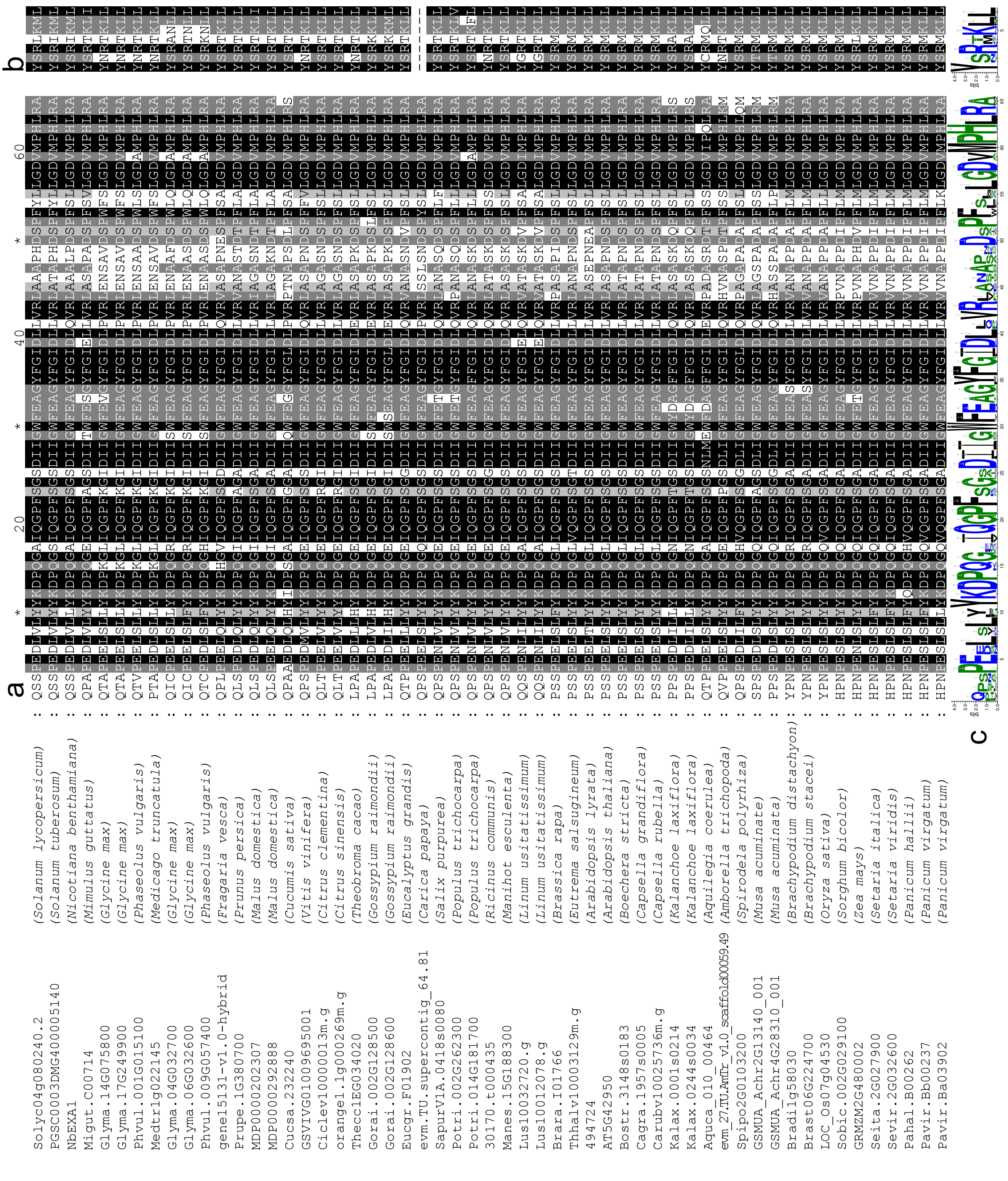

Supplementary Figure 3. Sequence analysis of EXA1 homologs

Protein sequence alignments of the (a) GYF domain and (b) eIF4E-binding motif of EXA1 homologs. (c) Consensus protein sequences of the GYF domain and eIF4E-binding motif of EXA1 homologs were drawn using the WebLogo 3 application (http://weblogo.threeplusone.com/).

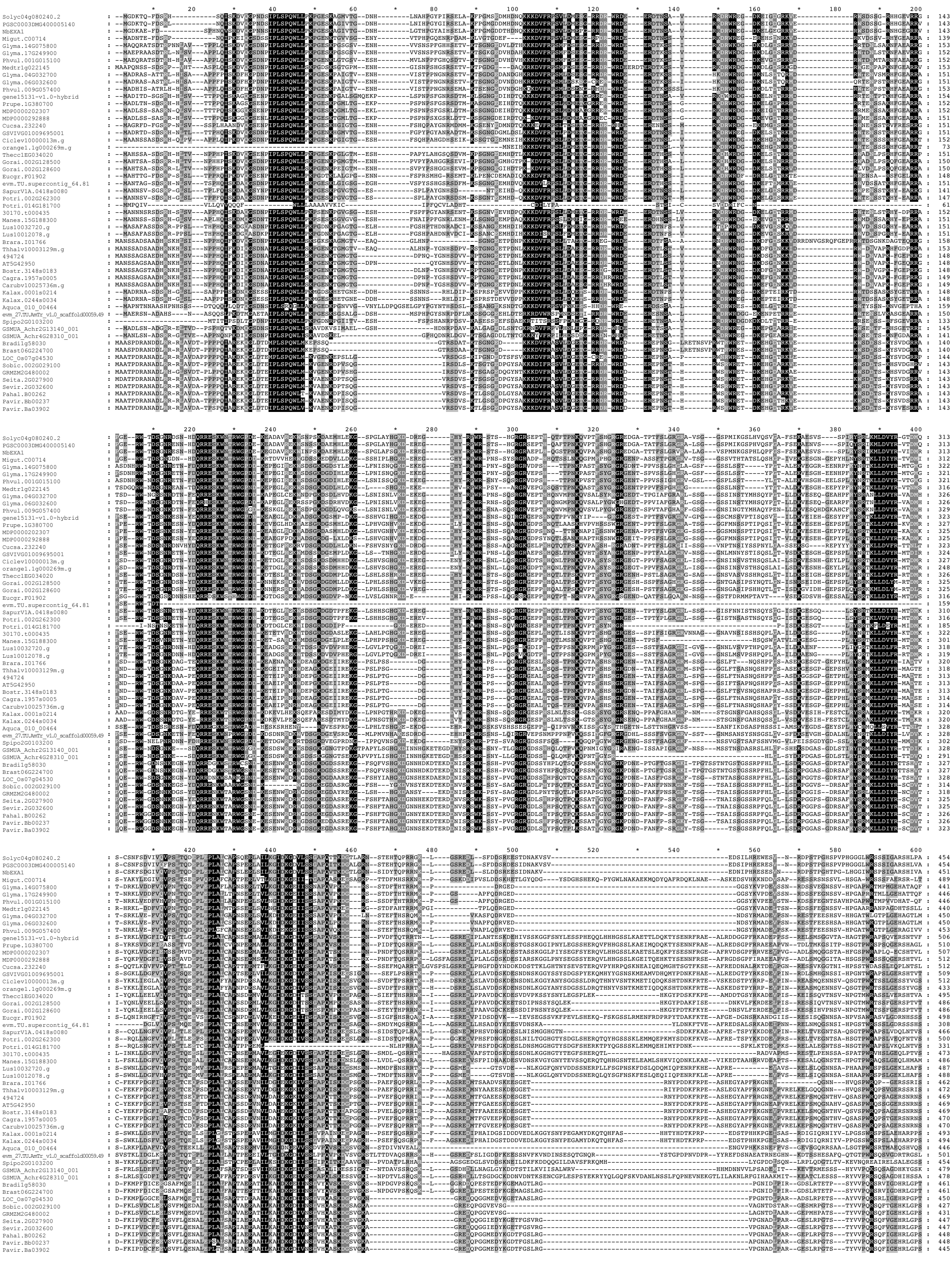

Supplementary Figure 4. Alignment of EXA1 homologous protein sequences

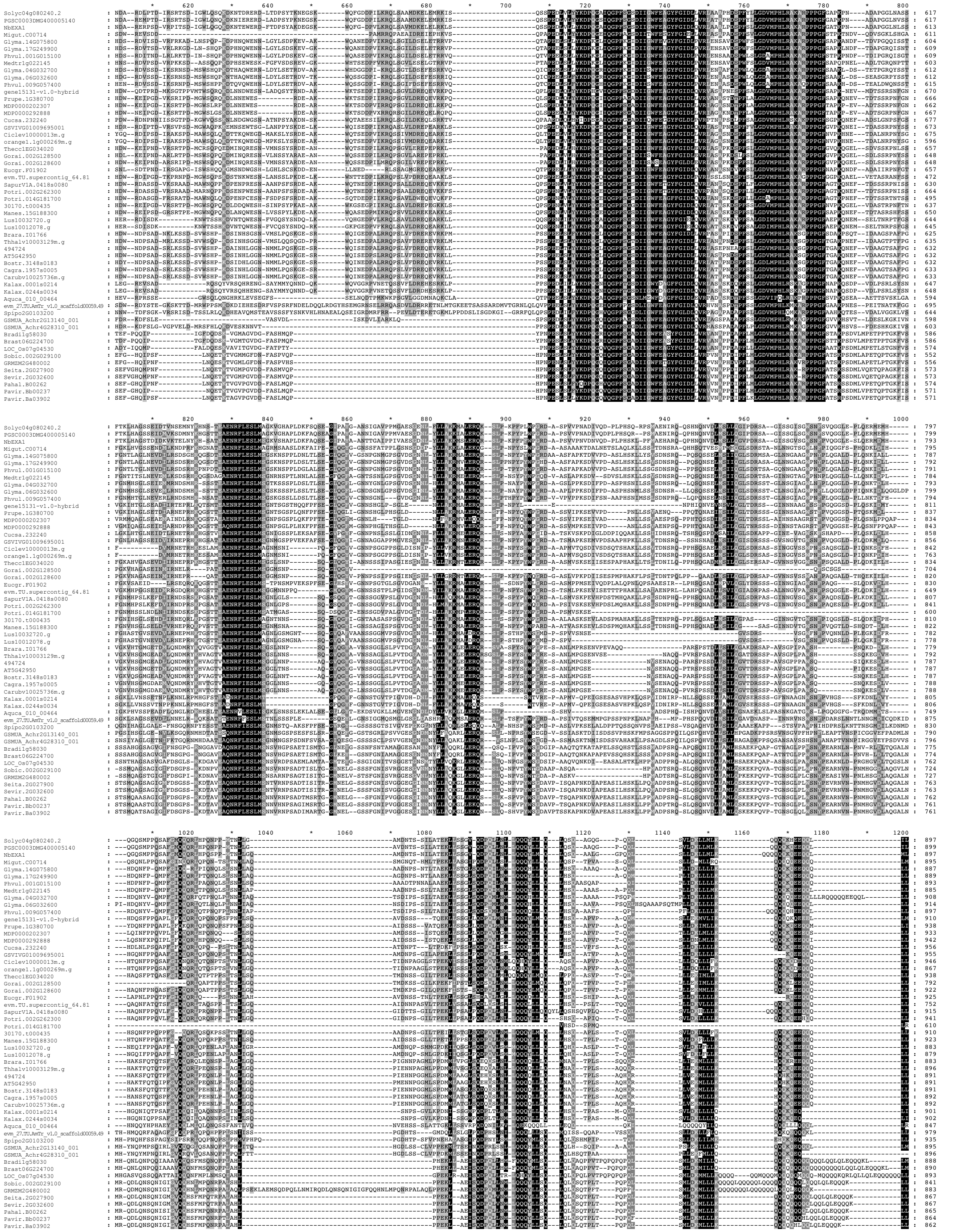

Supplementary Figure 4. (continued)

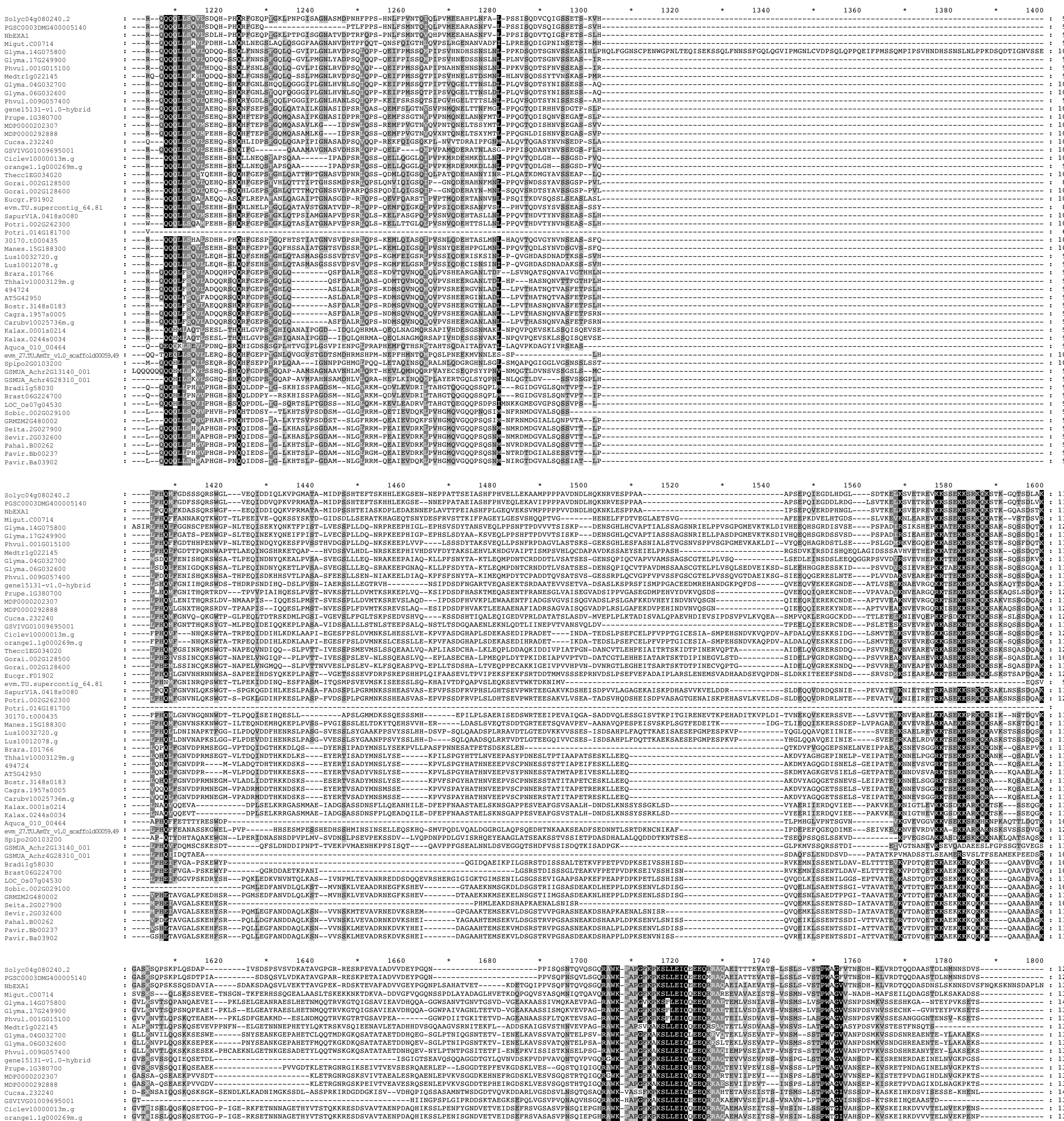

Supplementary Figure 4. (continued)

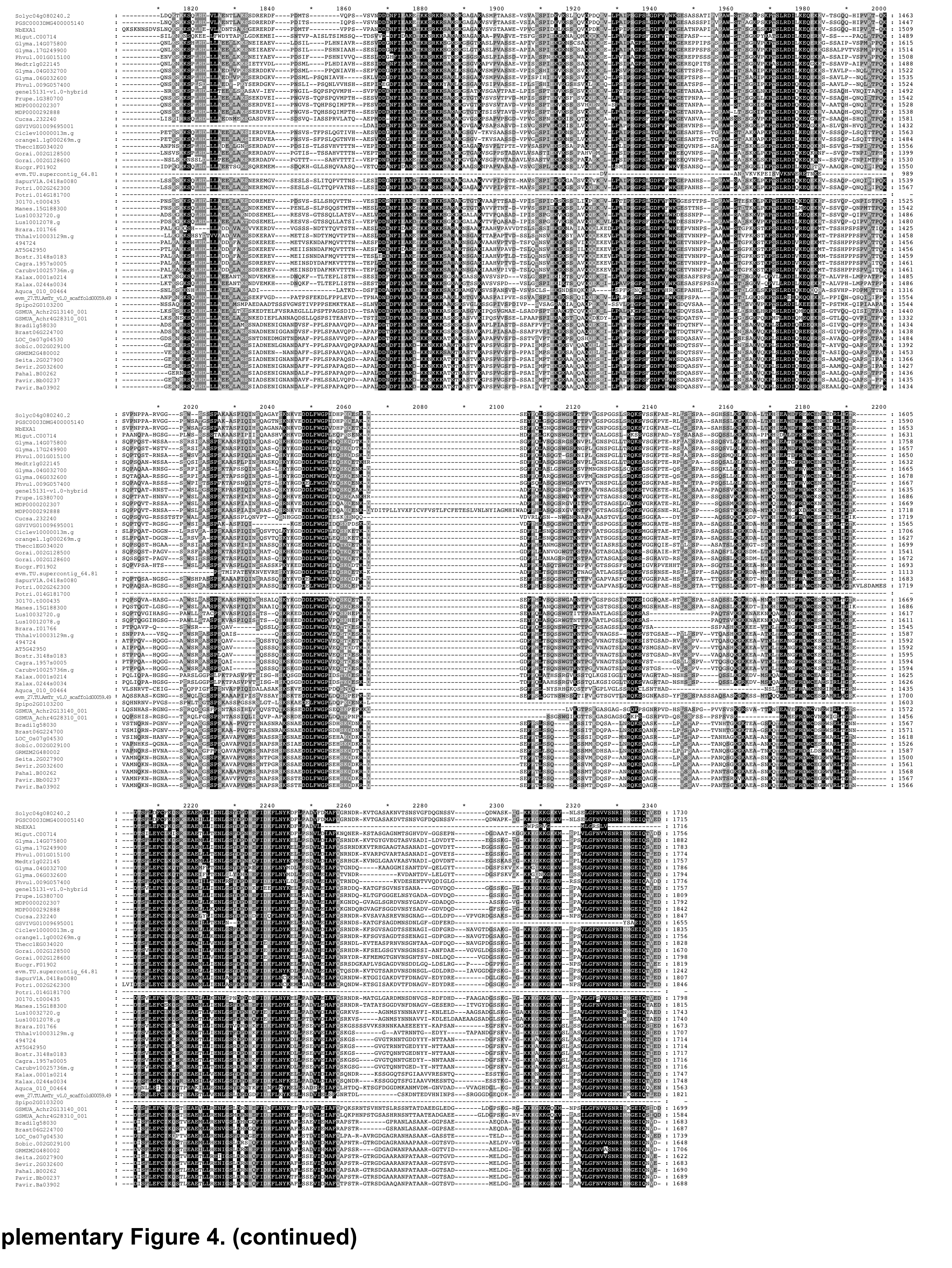

Supplementary Figure 4. (continued)

Supplementary Table 1. Primers used in this study

Primer Name	Sequence (5'-3')	Comments
qRT-PCR		
Nb18S-193F	ATACGTGCAACAAACCCCGAC	qRT-PCR for Nb18S rRNA
Nb18S-280R	TGAATCATCGCAGCAACGG	qRT-PCR for Nb18S rRNA
NbEXA1-rt12F	AAGTGGATGACGATCTGTTCTGG	qRT-PCR for NbEXA1 mRNA, and Detection for mRNA of NbEXA1
NbEXA1-rt12R	TCCTTTCACTGGTGTGGTCTTG	qRT-PCR for NbEXA1 mRNA
SlEXA1-rtF	TGGAGGACGATTTGTTTTGG	qRT-PCR for SlEXA1 mRNA
SlEXA1-rtR	TTCCCCAACTGCCTTGACTT	qRT-PCR for SlEXA1 mRNA
PlRep-F3	AATCCCCAGACTTCCATGAGCACC	qRT-PCR for PlAMV
PlRep-R3	TTTTCTTTGCGCCGAGCTTCTC	qRT-PCR for PlAMV
PVXCP-159F	CGCAACAAATGAGGACCTCAGCAAG	qRT-PCR for PVX-CP
PVXCP-239R	GCAGCCTGTGCCATAGTGTCTGTG	qRT-PCR for PVX-CP
WClMV-5636F	GTCCTTCCGAAGCTGAACTCTTAG	qRT-PCR for WClMV
WClMV-5717R	TTAGTGCCACGGCGTTTTG	qRT-PCR for WClMV
HdRSV-rt11F	ACAGTGGGTGAAGAAAATGGAGA	qRT-PCR for HdRSV
HdRSV-rt11R	CGGCGTGTTGTTGGAATG	qRT-PCR for HdRSV
PepMV-rtF	GACTTCTCAAATCCTAATACAGC	qRT-PCR for PepMV
PepMV-rtR	CACATCAGCATAAGCACGAGC	qRT-PCR for PepMV
CymMV-rt6F	CCCCGAGGATGTTATAGAAGGA	qRT-PCR for CymMV
CymMV-rt6R	GGTATCTGGTGGCGTTGTAGG	qRT-PCR for CymMV
AltMV-rt2280F	CCCCACTCCCTTTTCTCC	qRT-PCR for AltMV
AltMV-rt2245R	ATTGGCGTTGACCATTCTCC	qRT-PCR for AltMV
LoLV-rt7F	CAGCAATGCGAGGGACTATCTAC	qRT-PCR for LoLV
LoLV-rt7R	TGTCGGGGTTTGAGTTTGG	qRT-PCR for LoLV
PVM-rtF	GTCCCACCCCAAGAGAGAAG	qRT-PCR for PVM
PVM-rtR	TCAGCATTGAGCGAACTAAACAC	qRT-PCR for PVM
TMV-rt183K-2048F	CGGCAGATTCGTTAAATTCGT	qRT-PCR for YoMV
TMV-rt183K-2165R	GACACCGCAGCAGATAGTGA	qRT-PCR for YoMV
plasmid construction		
Xh-NbEXA1-268F	CCGCTCGAGGAACCCGGTCGACGTGACCG	Construction for pCAM-ALSV-NbEXA1, and PCR for DIG probe
Bm-NbEXA1-567R	CGCGGATCCTTTGCTAGGGTTGATCCATTTC	Construction for pCAM-ALSV-NbEXA1, and PCR for DIG probe
Xh-SlEXA1-865F	CCGCTCGAGGCTGAAAGTGTTTCCTCTCC	Construction for pTRV2-SlEXA1
Bm-SlEXA1-1365R	CGCGGATCCATTAGCCGGCAGATGTGAAC	Construction for pTRV2-SlEXA1
NbEXA1-F	GACTGGATCCGGTACATGGGTGACAAAGCTGAATTC	Construction for p35S-NbEXA1g
NbEXA1-R	TCTCGAGTGCGGCCGCTAATCTTCCACAGTCTGAATC	Construction for p35S-NbEXA1g
Kp-SlEXA1-5U-F	CGGGGTACCGACTTCTTCTCCCCTTCTTCCC	Construction for p35S-SlEXA1g, and Detection for mRNA of SlEXA1
Nt-SlEXA1-R	ATAGTTTAGCGGCCGCGAATGAACCCACCTCAGCTAAAG	Construction for p35S-SlEXA1g
Bm-OsEXA1-F	CGCGGATCCATGGCCGCCACTCCGGACCGCGCCAAT	Construction for p35S-OsEXA1g, and Detection for mRNA of OsEXA1
Nt-OsEXA1-R	ATAGTTTAGCGGCCGCGACTAATCTTCCACGTTCTGGATCTCA	Construction for p35S-OsEXA1g
AltMV-1F	GAAAAGTAAAGCAAAGCAAAGC	Construction for pCAM-AltMV
Alt35SR	GCTTTGCTTTGCTTTACTTTTCCCTCTCCAAATGAAATGAAC	Construction for pCAM-AltMV
CymMV-1F	GAAAACCAAACCTCACGTCT	Construction for pCAM-CymMV
CymMV-35S-R	AGACGTGAGGTTTGGTTTTCCCTCTCCAAATGAAATGAAC	Construction for pCAM-CymMV
Hd24F	GAAAAGTTCCACACCCAAACCAAA	Construction for pCAM-HdRSV
Hd35SR	GTTTGGGTGTGGAACTTTTCCCTCTCCAAATGAAATGAAC	Construction for pCAM-HdRSV
Lol-1F	GAAAACCAAACCAAACCACACCAGC	Construction for pCAM-LoLV
Lol-7650R-polyA40	TTTTTTTTTTTTTTTTTTTTTTTTTTTTTTTTTTTTTTTTGCTTTGACGGCAAAACCGAGGGTGT	Construction for pCAM-LoLV
20polyA-NOS	AAAAAAAAAAAAAAAAAAAACCGTTACGTAGCGGTACCCC	Construction for pCAM-LoLV
Lol35SR	GGTGTGGTTTGGTTTGGTTTTCCCTCTCCAAATGAAATGAAC	Construction for pCAM-LoLV
KpGR3nest	GGGGTACCGCTACGTAACGGCATGACAGTG	Construction for pCAM-AltMV, CymMV, HdRSV and LoLV
KpGR3nesF	CCGTTACGTAGCGGTACCCCTCAAACATTTGGCAATAAA	inverse-PCR for pCAMBIA1301
RT-PCR		
Nt-NbEXA1-5451R	ATAGTTTAGCGGCCGCGACTAATCTTCCACAGTCTGAATC	Detection for mRNA of NbEXA1
SlEXA1g-3301R	CGGATGTGCATTGAGATGGT	Detection for mRNA of SlEXA1
OsEXA1-1480R	GATCTTTGTAATATAAAGATAAG	Detection for mRNA of OsEXA1
TRV-F	GCTGCTAGTTCATCTGCAC	Detection for TRV
TRV-R	GCACGGATCTACTTAAAGAAC	Detection for TRV
Southern blot analysis		
NbEXA1g-5015F	TACAGAATGGGAAAGAGATTTC	PCR for GYF domain-specific DIG probe
NbEXA1g-5500R	GCCTCCGTTGCTGAATGC	PCR for GYF domain-specific DIG probe
